# Cardiac changes in children with post-streptococcal glomerulonephritis: a prospective observational study

**DOI:** 10.1186/s12887-026-07396-z

**Published:** 2026-09-28

**Authors:** Sherin Khamis Hussein, Moaz Yasser Darwish, Sara I. Abo Elnour

**Affiliations:** 1 Department of Pediatrics, Faculty of Medicine, Fayoum University, Fayoum, Egypt; 2 Faculty of Medicine, Fayoum University, Fayoum, Egypt

**Keywords:** Post-streptococcal Glomerulonephritis, PSGN, Myocardial impairment, Cardiac status, Myocardial performance index, MPI

## Abstract

**Background:**

Acute poststreptococcal glomerulonephritis (PSGN) is the most common type of glomerulonephritis which may involve multiple organs, including the heart. This study aimed to explore cardiac status of children with PSGN and track progression of any cardiac impairment using a comprehensive multimodal assessment approach.

**Methods:**

This observational study included 41 patients with PSGN (1–12 years) who underwent clinical, laboratory, electrocardiography, echocardiography and tissue Doppler assessment between January 2024 and April 2025 at Fayoum University Hospitals. Patients were followed up for 12 weeks after the diagnosis was established.

**Results:**

Prolonged QTc was reported in 13 cases (median=431ms), showing a significant negative correlation with ejection fraction (EF) (*r*=-0.40, *P* = 0.009) and fractional shortening (FS) (*r*=-0.38, *P* = 0.01) and a positive correlation with estimated systolic pulmonary artery pressure (ESPAP) (*r* = 0.36, *P* = 0.02). Ten patients (24.4%) had mild mitral regurgitation, and one (2.4%) had moderate regurgitation. At follow-up, cases showed a significant decrease in interventricular septum thickness (IVS), left ventricular end-diastolic diameter (LVEDD), and ESPAP with *P* = 0.003, 0.01, and 0.001, respectively. Thirty patients had high right ventricular myocardial performance index (MPI), and 17 had high left ventricular MPI at diagnosis. Significant decrease was observed in isovolumetric contraction time (IVCT) (RV: *P* = 0.03, LV *P* = 0.01) and MPI (*P* < 0.001 for both ventricles).

**Conclusion:**

Transient subclinical functional cardiac changes were evident in pediatric patients with PSGN. These transient changes may impose significant clinically relevant risks, particularly in the acute phase of the disease. Selected patients in appropriate clinical contexts may benefit from advanced cardiac investigations. QTc prolongation may warrant attention as an initial assessment tool of myocardial status, while myocardial performance index can be considered as an advanced measure. However, both tools should be interpreted with caution and within the relevant clinical context since they are subjected to various confounders.

**Supplementary Information:**

The online version contains supplementary material available at https://doi.org/10.1186/s12887-026-07396-z.

## Introduction

Acute Poststreptococcal glomerulonephritis (PSGN) is known as the leading cause of kidney injury in children, especially in developing countries [1, 2]. The annual incidence of PSGN in developing countries is estimated to range between 8.5 and 28.5 cases per 100,000 individuals [2]. 

PSGN classically presents with the triad of nephritic syndrome: hematuria, edema, and hypertension [3, 4]. Beyond this presentation, PSGN can trigger multiorgan involvement that can include heart, lung and brain [5, 6]. Cardiac involvement represents a serious complication that may manifest as congestive heart failure with pulmonary edema as a consequence of hypervolemia [5]. In addition, direct immune-mediated myocardial involvement has been also described [7].

Despite nephritic syndrome being the most common cause of hypertensive heart failure in the pediatric population [8], studies that evaluate cardiac status in children suffering from this condition remain scarce [6, 8]. Although echocardiography has become more accessible, it is still rarely utilized in the assessment of PSGN, leaving subclinical involvement undetected [9]. Moreover, previous studies adopted conventional echocardiography rather than advanced assessment methods such as tissue Doppler imaging and Myocardial Performance Index (MPI). We hypothesized that subclinical cardiac involvement could be present in children with PSGN and detectable using such advanced tools. Therefore, this exploratory study aimed to explore cardiac status of children with PSGN and track progression of any cardiac impairment using a comprehensive multimodal assessment approach.

## Methods

### Study setting and subjects

This prospective observational study included 41 children aged 1 to 12 years who were diagnosed with PSGN and admitted to Fayoum University Hospital. All patients were admitted for 5 to 7 days then discharged to continue regular follow-up at the nephrology outpatient clinic of the pediatric department in collaboration with the cardiology unit. Children with heart disease, pulmonary disease, urinary tract anomalies, or other causes of nephritis were excluded. During admission patients received furosemide to attain euvolemia and to control blood pressure and those who did not show an adequate response were given nifedipine. All study participants received amoxicillin treatment. Cardiac changes were assessed in all patients through a multimodal approach encompassing ventricular dimensions, systolic function (EF, FS), diastolic indices, MPI, and ECG parameters.

### Clinical and laboratory assessment

All patients underwent clinical assessment, including medical history and examination. Oliguria was defined as urine output of less than 0.5 mL/kg/hour. Blood pressure was measured in the right arm at rest using an appropriately sized cuff. Anemia was defined according to the World Health Organization (WHO) cut-off criteria [10]. Patients also underwent laboratory investigations, including complete blood count, urea, creatinine, C-reactive protein (CRP), Erythrocyte sedimentation rate (ESR), Antistreptolysin O Titer (ASOT) and complement proteins C3 and C4. Diagnosis of PSGN was established based on the presence of nephritic syndrome with a history of hematuria and elevated ASOT, supported by consumed C3 when present. One patient with normal C3 levels was included based on strong clinical suspicion, having fulfilled all other diagnostic criteria including nephritic syndrome, hematuria, and elevated ASOT.

### Echocardiographic evaluation

Echocardiography was performed by an experienced pediatric cardiologist using a Vivid 5 color Doppler ultrasound system (General Electric) with transducers of 3.75 MHz or 5 MHz, as appropriate for children or adolescents. A comprehensive echocardiographic examination was performed to exclude the possibility of congenital heart disease and assess cardiac function. This assessment included the evaluation of global function, left (LV) dimensions along with LV function. Using the standard transthoracic windows, LV end diastolic diameter (LVEDD), LV end systolic diameter (LVESD), LV posterior wall (LVPW). Additionally, transmitral E and A waves velocity were obtained. Echocardiographic assessment was conducted during the admission period and repeated 12 weeks later. Normal ejection fraction (EF) was concluded whenever it was ≥ 55%.

### Myocardial Performance Index (MPI)

MPI was calculated as the sum of isovolumetric relaxation time (IVRT) and isovolumetric contraction time (IVCT), divided by the ejection time (ET), (MPI = (IVCT + IVRT) / ET). In the absence of universally established pediatric reference ranges for MPI, upper and lower normal limits were approximated using mean ± 2 standard deviations derived from Eidem et al. 2004, which assessed MPI in healthy children. Eidem et al. provided mean and standard deviations for different pediatric age groups which allowed for age-adjusted cutoff values [11].

### Electrocardiographic assessment

We examined 12-lead ECG recordings on admission (using Cardiofax ECG-9620 L, a real-time electrocardiograph with a 6-channel recorder) at a speed of 25 mm/s and amplitude of 1 mV/cm. We measured heart rate, QRS axis, T axis, QRST angle, P axis, P-R interval, QRS duration, QT, QTc measured, ST segment, and T wave. We selected the leads with the longest QT interval duration in leads II and V5. The Bazett method was adopted to compute the corrected QT interval (QTc) and QTc values of more than 460ms.were considered frankly prolonged, while values of (440-460ms) were considered borderline [12, 13]. ECG assessment was conducted during the admission period and repeated 12 weeks later.

### Ethical considerations

The study was carried out according to the principles of the Declaration of Helsinki and its appendices and was approved by the hospital ethical review board in Fayoum University Hospital, number R658. Written informed consents from patients’ caregivers were obtained for the use of their study-related information and for participation in the ongoing research.

### Statistical methods

Data analysis was performed using the Statistical Package of Social Science (SPSS) software version 22 for windows 7 (SPSS Inc., Chicago, IL, USA). Simple descriptive analysis was provided for qualitative and quantitative data. Qualitative data were presented in the form of frequencies and percentages. Quantitative data were presented using means, medians, standard deviations (SD), and ranges.

All quantitative data were first tested for normality using the one-sample Kolmogorov-Smirnov test prior to selecting inferential statistical tests. Regarding parametric quantitative data, independent samples t test was used to compare between independent outcome measures while paired t-test was adopted for dependent quantitative data. Bivariate Pearson correlation test was adopted to examine the association between quantitative parametric variables. For categorical variables, which were summarized as frequencies and proportions, the Chi-square test was used for comparisons between independent groups, whereas the **McNemar test** was applied for paired dependent categorical data. Effect size was calculated by Cohen’s d for t-test and Cramer’s V for chi square test. P values of less than 0.05 was considered significant.

## Results

Demographic data of the patients is shown in Table 1; the mean age among the study group was 7.5 ± 3.1 years old, ranging between 1 and 12 years, with 30 patients (73.2%) being males and 11 patients (26.8%) being females.

**Table 1:** Description of demographic and clinical assessment among nephritis cases

Variables	Demographic and clinical data (*n* = 41)	
	Mean ± SD	Median (Range)
Age (years)	7.5 ± 3.1	8(1–12)
Sex	**No.**	**%**
Male	30	73.2%
Female	11	26.8%
Medical history	**No.**	**%**
Lower limb edema	14	34.1%
Facial oedema	25	61%
History of URTI	33	80.5%
Oliguria	28	68.3%
Hematuria	41	100%

Vital signs	**Mean ± SD**	**Median (Range)**
SBP (mmHg)	109.6 ± 15.6	105(88–160)
DBP (mmHg)	69.4 ± 15.6	60(50–117)
HR (beats/minute)	95 ± 19	100(54–135)
RR (breaths/minute)	23 ± 2	23(20–29)

*SBP* Systolic blood pressure, *DBP* Diastolic blood pressure, *HR* Heart rate and *RR* Respiratory rate

**No**. indicates that the number of patients who experienced a specific event is listed below, **% **indicates that the percentage of patients who experienced a specific event is listed below

Clinically, all of the included children had hematuria, 28 patients (68.3%) showed oliguria, 25 patients (61%) had periorbital oedema and 14 patients (34.1%) had lower limb edema. Thirty-three patients (80.5%) reported a history of upper respiratory tract infection (URTI).

Notably, the youngest patient in the cohort was one year old. Although PSGN is uncommon below two years of age, the diagnosis in this case was established based on typical clinical assessment and laboratory findings. The patient’s family reported a history of previous URTI and reported the presence of hematuria. Clinical examination revealed hypertrophied tonsils and hematuria was confirmed in the inpatient setting. The patient’s laboratory findings exhibited elevated ASOT and consumed C3. Anti-double-stranded DNA and antinuclear antibody were negative. The patient’s family denied any family history of renal disease. Urological causes of hematuria were excluded.

According to the laboratory findings of the patients shown in Table 2, most of the patients had mild anemia, normal white blood cell count (WBC), and Platelets (PLT) counts. They also showed normal albumin levels and electrolytes with high levels of antistreptolysin O titer (ASOT). All cases had normal arterial blood gas (ABG) analysis. The majority of cases showed high CRP (78%), with 40 cases (97.6%) showing consumed C3 levels. All 41 patients showed normal levels of C4.

**Table 2:** Description of laboratory investigations among nephritis cases

Variables	Number (*n* = 41)	
	Mean ± SD	Median (Range)
Urine output (mL/kg/hour)	1.98 ± 0.45	2.1(0.9–2.6)
HB (g/L)	10.6 ± 1.05	10.3(8.7–12.9)
WBC (×10³/µL)	9.9 ± 4.5	8.8(3–23)
PLT (×10³/µL)	340.8 ± 81.7	342(193–498)
ESR (mm/hour)	25.7 ± 8.4	20(19–44)
CRP (mg/L)	33.2 ± 24.4	22(12–90)
Albumin (g/dL)	3.9 ± 0.37	4(2.9–4.5)
Urea (mg/dL)	61 ± 41.3	37(22–168)
Creatinine (mg/dL)	1.03 ± 0.82	0.9(0.3–4.1)
Na (mEq/L)	139.5 ± 4.2	139(131–149)
K (mEq/L)	4.9 ± 0.81	4.9(3.3-6)
C3 (mg/dL)	26.9 ± 14.3	28(13–96)
C4 (mg/dL)	30.3 ± 8.9	32(15–44)
ASOT (IU/mL)	490.8 ± 161.5	400(323–1010)
ABG		
PH	7.3 ± 0.06	7.3(7.3–7.44)
CO2 (mEq/L)	33.9 ± 4.02	33(30–39)
HCO3 (mEq/L)	23.9 ± 4.7	25(18–30)
CRP	**No.**	**%**
Negative	9	22%
Positive	32	78%
C3 level		
Consumed	40	97.6%
Normal	1	2.4%
C4 level		
Consumed	0	0%
Normal	41	100%

*HB* Hemoglobin, *WBC* White blood cell count, *PLT* Platelets, *ESR* Erythrocyte sedimentation rate, *CRP* C-reactive protein, *Na* Sodium, *K* potassium, *C3* Complement component 3, *C4* Complement component 4, *ASOT* Antistreptolysin o titer, *ABG* Arterial blood gas, *PH* Potential of hydrogen, *CO2* Carbon dioxide and HCO3: bicarbonate

**No**. indicates that the number of patients who experienced a specific event is listed below, **% **indicates that the percentage of patients who experienced a specific event is listed below

Regarding cardiac assessment by echocardiography and tissue Doppler (Tables 3 and 4, respectively) at the time of diagnosis, all patients had normal conventional systolic function as evidenced by normal ejection fraction and fractional shortening. However, tissue Doppler imaging revealed subclinical myocardial changes in many patients, with 30 (73.2%) patients demonstrating elevated RV MPI and 17 (41.5%) patients demonstrating elevated LV MPI. At 12 weeks, five (12.2%) and two (4.9%) patients had elevated RV MPI and LV MPI, respectively. Ten patients (24.4%) having functional mild mitral regurgitation and one patient (2.4%) having moderate regurgitation. At follow-up time, cases enrolled in the current study show a significant decrease in IVS, LVEDD, and ESPAP measures with *P* values of 0.003, 0.01, and 0.001, respectively. In addition, there was a significant improvement in mitral regurgitations at follow-up with a *P* value < 0.001. No statistically significant difference was observed between males and females as regards other echo and tissue Doppler measurements.

**Table 3:** Comparison of Echo finding at the diagnosis and follow up among nephritis cases

Variables	Echo findings		*P* value	95% CI		Effect size
	Baseline	Follow-up				
	Mean ± SD	Mean ± SD		Lower	Upper	
AO (cm)	2.2 ± 0.36	2.2 ± 0.36	0.36	0.04 -	0.01	0.0
LA (cm)	2.4 ± 0.49	2.4 ± 0.49	0.57	-0.012	0.022	0.0
IVS (cm)	0.79 ± 0.16	0.76 ± 0.13	0.003*	0.009	0.04	0.21
LVPW (cm)	0.72 ± 0.19	0.72 ± 0.18	0.99	-0.01	0.012	0.0
LVEDD (cm)	3.7 ± 0.59	3.6 ± 0.54	0.01*	0.007	0.07	0.18
LVESD (cm)	2.3 ± 0.44	2.3 ± 0.44	0.23	-0.008	0.03	0.0
EF (%)	67.8 ± 5.6	68.1 ± 4.4	0.47	-1.29	0.61	0.06
FS (%)	36.9 ± 3.8	37.3 ± 2.9	0.27	-1.03	0.30	0.12
ESPAP (mmHg)	28.3 ± 7.7	25.8 ± 5.6	0.001*	1.1	3.9	0.37

Mitral regurgitation	No. (%)	No. (%)				
Normal mitral valve without regurgitation	30 (73.2%)	40 (97.6%)	< 0.001*	0.02	0.03	0.37
Normal mitral valve morphology with mild MR	10 (24.4%)	-----				
Moderate MR	1(2.4%)	1(2.4%)				

*AO* Aortic annulus, *LA* Left atrium, *IVS* Interventricular septum, *LVPW* Left ventricular posterior wall, *LVEDD* Left ventricular end diastolic diameter, *LVESD* Left ventricular end systolic diameter, *EF* Ejection fraction, *FS* Fractional shortening, *ESPAP* Estimated systolic pulmonary artery pressure and *MR* Mitral regurgitation, *P *values reported in bold and followed by "*" indicate significance

**Table 4:** Comparison of tissue doppler findings at baseline and follow up among nephritis cases

Variables	Tissue Doppler findings		*P* value	95% CI		Effect size
	Baseline	Follow-up				
	Mean ± SD	Mean ± SD		Lower	Upper	
Right ventricle						
IVCT (ms)	52.6 ± 8.4	49.1 ± 5.9	0.03*	0.29	6.7	0.48
IVRT (ms)	88.7 ± 20.6	82.5 ± 13.8	0.07	-0.60	13.1	0.35
ET (ms)	276.7 ± 45.6	314.3 ± 24.4	< 0.001*	-52.6	-22.7	1.03
RV MPI	0.51 ± 0.09	0.40 ± 0.05	< 0.001*	0.07	0.13	1.51
Left ventricle						
IVCT (ms)	51.6 ± 8.5	48.3 ± 5.1	0.01*	0.58	6	0.47
IVRT (ms)	86.8 ± 23.7	79.7 ± 13.1	0.08	-1.09	2.5	0.37
ET (ms)	292.6 ± 39.8	306.7 ± 26.1	0.09	-30.6	2.5	0.42
LV MPI	0.47 ± 0.08	0.41 ± 0.04	< 0.001*	0.03	0.08	0.95
TAPSE (mm)	1.69 ± 0.27	1.74 ± 0.23	0.02*	-0.08	-0.005	0.20
E/A ratio	1.98 ± 0.39	1.97 ± 0.33	0.86	-0.05	0.06	0.11
S-prime velocity (m/s)	0.06 ± 0.004	0.06 ± 0.004	0.32	-0.00025	0.0007	0
E-prime velocity (m/s)	0.06 ± 0.009	0.06 ± 0.009	0.32	-0.001	0.003	0
A-prime velocity (m/s)	0.05 ± 0.003	0.05 ± 0.005	0.32	-0.001	0.003	0
E/E-prime ratio	10.3 ± 1.8	10.1 ± 1.8	0.21	-0.12	0.52	0
Deceleration time (ms)	108.6 ± 10.9	108.5 ± 11.1	0.32	-0.12	0.36	0

*IVCT* Isovolumetric contraction time, *IVRT* Isovolumetric relaxation time, *ET* Ejection time, *RV MPI* Right ventricular myocardial performance index, *LV MPI* Left ventricular myocardial performance index, *TAPSE* Tricuspid annular plane systolic excursion, *E/A* Early (E) to late (A) ventricular filling velocities S-prime, systolic myocardial velocity, E-prime velocity: Early diastolic myocardial velocity, *A-prime velocity* Late diastolic myocardial velocity, *E/E-prime ratio* Ratio of early transmitral flow velocity (E) to early diastolic myocardial velocity (E-prime), *mm* Millimetres, *m/s* Meter per second and ms: millisecond, *P *values reported in bold and followed by "*" indicate significance

At follow-up time, cases enrolled in the current study show a statistically significant decrease in IVCT (*P* = 0.03 and LV: *P* = 0.01) and MPI (*P* < 0.001 for both ventricles) in tissue Doppler findings. In addition, there was a statistically significant increase in ET (*P* < 0.001) on the right side and TAPSE (*P* = 0.02) measure at follow-up (Table 4).

Regarding the ECG illustrated in Table 5, Fifteen patients had notable QTc values, 13 (31.7%) with frank prolongation (QTc > 460 ms) and two (4.9%) with borderline values (QTc 440–460 ms). The remaining 26 patients had normal QTc values. The median QTc across the entire cohort was 431 ms (range 390–500 ms). 

No patient had a history of medications known to prolong QTc. QTc showed a statistically significant negative correlation with EF and FS measures (*r*=-0.40, *P* = 0.009 and *r*=-0.38, *P* = 0.01, respectively) and a positive correlation with ESPAP measure (*r* = 0.36, *P* = 0.02). A statistically significant difference was observed regarding EF between patients with frankly prolonged QTc (> 460 ms) and patients with normal or borderline QTc (≤ 460 ms) with a p-value of 0.04 (Supplementary Table 1). No significant correlation was observed between QTc and other echocardiography findings, laboratory parameters, SBP or DBP. Normal sinus rhythm was evident in all patients.

**Table 5:** ECG findings in the studied patients at baseline

Variables	ECG findings	
	Mean ± SD	Median (Range)
Heart rate	90 ± 19.4	95(54–120)
p-wave axis	32.9 ± 24.9	32(0–85)
QRS axis	55.7 ± 26.1	63(8–97)
T-wave axis	51.6 ± 23.4	53(7-114)
PR interval	92.2 ± 21.05	86(52–130)
QRS duration	69.8 ± 6.9	70(58–80)
QT duration	362.5 ± 30.9	360(316–438)
QT corrected	437.2 ± 32.1	431(390–500)
Rhythm	No. (%)	
Sinus	41 (100%)	

## Discussion

The current study aimed to explore cardiac status of children with PSGN and track progression of any cardiac impairment using a comprehensive multimodal assessment approach that included electrocardiography, echocardiography and advanced tissue Doppler imaging measurement. Transient subclinical functional cardiac changes were evident in 30 (73.2%) and 17 (41.5%) patients regarding right and left ventricles, respectively, evidenced by high MPI for age. This finding demonstrates that cardiac changes may be present in pediatric patients with PSGN but detectable only using advanced cardiac assessment tools. A statistically significant improvement in MPI was observed after 12 weeks, which supports reversibility of these cardiac changes. MPI is an important measure of left and right ventricular function which offers a doppler assessment of combined systolic and diastolic cardiac performance. It is also considered as a predictive indicator of congestive heart failure [14, 15]. The adoption of MPI in this context represents a novel contribution to the growing body of literature on cardiac complications of PSGN in children.

The QT interval in ECG is usually corrected to heart rate, yielding the QT-corrected time (QTc). In children 1–15 years of age, a QTc > 440 ms is considered to be the borderline upper limit of the QTc interval, while QTc > 460 ms is considered prolonged [13]. In the current study, 13 patients (31.7%) were considered to have frankly prolonged QTc and two patients (4.9%) had borderline QTc. This finding is comparable with a previous finding reported by Idhate et al., 2017 [6], where prolonged QTc was identified in 11 out of 30 children with PSGN (36.6%). A previous randomized controlled study [8] reported that 22 out of 60 children with acute nephritic syndrome (37%) had prolonged QTc, which goes in alignment with our findings as well. QT prolongation carries high risk of ventricular arrythmias and sudden cardiac death [16]. QTc showed significant correlations with EF, FS, and ESPAP across the entire cohort. Also, a significant difference was observed in terms of EF between patients with frank QTc prolongation and others with borderline or normal QTc (*P* = 0.04). These findings suggest that QTc can be considered as one of the initial cardiac assessment measures that guides physicians in the process of identifying patients who need further advanced cardiac evaluation. Further validation of QTc as a potential simple, widely available and non-invasive initial assessment tool of myocardial status in PSGN is required in future larger, controlled prospective studies that assess the diagnostic utility.

Various factors can induce QTc prolongation in the context of PSG, including hypervolemia, renal impairment or electrolyte disturbances [6]. Also, QTc prolongation can be a manifestation of pre-existing conditions. Therefore, future studies should also consider serial ECG measures to confirm the relationship between QTc interval changes and disease context.

In a study by Idhate et al., 2017 [6], hypertension was identified as the most common cause of myocardial dysfunction in PSGN patients. On the other hand, our study did not find a statistically significant correlation between either systolic or diastolic function and SBP. Additionally, no significant correlation was observed between SBP or DBP and MPI or QTc interval which suggests that cardiac changes in PSGN is multifactorial rather than solely attributed to hypertension. Furthermore, the myocardial response to hypertension or pressure overload, such as hypertrophy and remodeling, develops over weeks to months of sustained hypertension, which contrasts with the acute nature of PSGN [17].

Apart from hypertension, other probable explanations of cardiac changes in these cases include, electrolyte disturbances, hypervolemia (especially when anuria or oliguria is present) and immune-mediated myocardial damage [18]. Imbalances in key electrolytes, particularly potassium, may affect the cardiac action potential, which can be reflected on parameters such as QTc interval and MPI [19]. In the current study, RV MPI and QTc interval showed no significant correlation with potassium and sodium levels. Similarly, LV MPI showed no significant correlation regarding potassium; however, a weak positive correlation was noted with sodium levels (*r* = 0.37, *p* = 0.01). These findings suggest that electrolyte disturbances cannot be considered as the sole contributing factor to the observed cardiac alterations in our cohort.

Hypervolemia leads to increased preload, which can prolong IVCT and IVRT, leading to increase in MPI [20]. In addition to hypervolemia, inflammatory response can serve as another explanation of the cardiac changes. The pathophysiology of the cardiac inflammatory response in PSGN is still unclear due to the presence of various streptococcal strains. The pathogenic antigen (SpeB proteinase) interestingly causes endothelial cells, cardiac myosin, and valvular tissue to develop cross-reactive antibodies. Mechanisms such as B cell reactivity, antibody deposition, and molecular mimicry produce valvulitis. Volume overload, angiotensin system and cardiorenal responses may further amplify the myocardial impairment [21, 22]. The normal EF of the current cohort, along with the elevated MPI that significantly improved after 12 weeks may suggest volume-mediated dysfunction as the predominant mechanism. However, both transient mild volume-mediated dysfunction or mild subclinical myocarditis can be considered as potential explanations and further distinction is required in future studies. Moreover, the significant decrease in IVS, LVEDD and ESPAP at follow-up further support the reversibility of the myocardial alterations observed in the early phase of PSGN.

### Strength and limitations

This observational study offers a prospective evaluation of cardiac changes in patients with PSGN after exclusion of pre-existing cardiac conditions. The adoption of various cardiac assessment tools is considered a key strength point of the current work. The comprehensive, multimodal approach integrated clinical, laboratory, echocardiographic, and ECG parameters into findings interpretation. This allowed for detailed cardiac function characterization. Although based on a single-center cohort of 41 patients, this study represents a novel contribution to understanding myocardial changes in pediatric PSGN. Regarding QTc prolongation, the absence of serial ECG measures limits the ability to rule out pre-existing conditions affecting QTc; therefore, future researchers should consider serial measures. The adoption of MPI in this context helped identify myocardial changes that were previously unrecognized by conventional echocardiographic parameters. The adoption of mean ± 2 standard deviations provided by Eidem et al. 2004 [11] to approximate MPI cutoff values is subjected to inter-study variability, which should be considered when interpreting these findings. However, it was necessary to allow for consistent classification of our cohort. Also, the single-center approach may limit the generalizability of findings to more diverse populations. The use of Bazzett correction enhanced comparability with prior studies. However, it may be affected by heart rate variability in pediatric populations, which could influence the generalizability of QTc estimates.

The absence of a healthy control group limited the ability to directly compare cardiac parameters with those of age-matched healthy children. Therefore, future multi-center-controlled studies are recommended. Furthermore, despite the potential multi-factorial nature of the myocardial changes, the distinction between volume overload and immune-mediated myocardial changes requires further investigations. Future studies should therefore include biomarkers of volume status and other relevant cardiac or inflammatory markers to better understand the underlying mechanisms. The absence of ambulatory blood pressure monitoring is considered as another limitation since it offers a more precise characterization of the blood pressure status and its potential contribution to cardiac changes. Despite the fact that all echocardiographic examinations were performed by a single experienced pediatric cardiologist, which minimizes interobserver variability, formal intraobserver reproducibility was not assessed in the current study. Future studies should incorporate repeated measurements to assess intraobserver variability.

## Conclusion

Transient subclinical functional cardiac changes were evident in pediatric patients with PSGN. These transient changes may impose significant clinically relevant risks, particularly in the acute phase of the disease. Routine advanced cardiac investigations are not recommended in all PSGN cases; however, selected patients in appropriate clinical contexts may benefit from advanced assessment tools. The prolongation of Corrected QT interval may warrant attention as a potential simple, widely available and non-invasive initial assessment tool of myocardial status in PSGN, pending further validation in larger, controlled studies. The myocardial performance index can be considered as an advanced measure to detect myocardial changes that is unrecognized by conventional measures. However, both tools are subjected to various confounders; therefore, they should be interpreted with caution and within the relevant clinical context.

## Supplementary Information

Supplementary Material 1.

## Data Availability

All the incorporated data within the current research work will be available upon reasonable request.

## Abbreviations

PSGN: Poststreptococcal glomerulonephritis
LV: Left ventricle
FS: Fraction shortening
CRP: C reactive protein
TAPSE: Tricuspid annular plane systolic excursion
TV: Tricuspid valve
LVIDD: Left ventricular internal diameter end diastole
LVISD: Left ventricular end systole
MPI: Myocardial performance index
URTI: Upper respiratory tract infection
