# Supplementary material for: Cardiac changes in children with post-streptococcal glomerulonephritis: a prospective observational study
Source: BMC Pediatr. 2026 Sep 28;26:925. doi: 10.1186/s12887-026-07396-z (PMC13625491; doi:10.1186/s12887-026-07396-z)
Supplement: Supplementary file 1 [file 12887_2026_7396_MOESM1_ESM.docx]

**Supplementary Table 1: Comparison between high and normal QT corrected**

| **Variables** | **QTC** | | **p-value** | **95% CI** | | **Effect size** |
| --- | --- | --- | --- | --- | --- | --- |
|  | **≤460**  **(n=28)** | **>460 (n=13)** |  |  |  |  |
|  | **Mean ±SD** | **Mean ±SD** |  | **L** | **H** |  |
| LVEDD | 3.7±0.52 | 3.6±0.74 | 0.71 | -0.33 | 0.49 | 0.15 |
| EF | 68.9±5.9 | 65.2±3.9 | **0.04*** | 0.05 | 7.4 | 0.74 |
| RV PV MPI | 0.51±0.09 | 0.49±0.08 | 0.60 | -0.05 | 0.07 | 0.23 |
| LV PV MPI | 0.47±0.07 | 0.46±0.09 | 0.73 | -0.04 | 0.07 | 0.12 |
| LA | 2.38±0.44 | 2.36±0.63 | 0.90 | -0.32 | 0.36 | 0.04 |
| **Laboratory investigations** |  |  |  |  |  |  |
| Creatinine level | 1.06±0.88 | 0.96±0.67 | 0.73 | -0.46 | 0.65 | 0.13 |
| Na | 139.8±4.2 | 139.1±4.3 | 0.64 | -2.2 | 3.5 | 0.16 |
| K | 4.8±0.74 | 4.9±0.95 | 0.68 | -0.66 | 0.44 | 0.11 |

Effect size calculated by **(Cohen's d)** for t-test
